# Game on: immersive virtual laboratory simulation improves student learning outcomes & motivation

**DOI:** 10.1002/2211-5463.13567

**Published:** 2023-02-12

**Authors:** Danielle Tsirulnikov, Celeste Suart, Ream Abdullah, Felicia Vulcu, Caitlin E. Mullarkey

**Affiliations:** ^1^ Bachelor of Health Sciences Program, Faculty of Health Science McMaster University Hamilton ON Canada; ^2^ Department of Biochemistry and Biomedical Sciences McMaster University Hamilton ON Canada

**Keywords:** gamified interventions, laboratory simulations, learning outcomes, motivation, undergraduate education, virtual reality

## Abstract

The use of gamified learning interventions is expanding in postsecondary education as a means to improve students' motivation and learning outcomes. Virtual laboratory simulations have been used in science education to supplement students' learning, as well as to increase engagement with course material. Due to COVID‐19, many instructors sought to replace or supplement hands‐on ‘wet‐lab’ work in an online environment. In this paper, we explored how the use of head‐mounted display technology in two laboratory simulations impacts learner motivation and learning outcomes. We used a mixed‐methods approach to analyze the experience of 39 undergraduate participants, examining test scores pre‐ and postsimulation, qualitative feedback, and quantitative experience ratings. The head‐mounted display technology was described as easy to use, with eye strain identified as a common occurrence. Participants had increased test scores following the laboratory simulations, with no significant difference between simulation groups. Very positive self‐reported measures of motivation and learner engagement were documented. Ninety‐one percent of participants agreed that virtual reality laboratory simulation would be a good supplement to regular teaching modalities. Overall, our results suggest that immersive virtual reality laboratory simulations experienced through head‐mounted display technology can be used to enhance learning outcomes and increase learner motivation.

## Graphical abstract

We examined how immersive virtual reality laboratory simulations impact learner motivation and learning outcomes. Our results demonstrate that learners had increased test scores and very positive self‐reported ratings of motivation after simulation completion. Learners found the technology intuitive, although eye strain was reported. We identify best practices for implementation and suggest how simulations could supplement traditional laboratory teaching modalities.

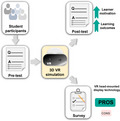

**Abbreviations**
ROUT: robust regression and outlier removal
VR: virtual reality

Motivation is a key determinant of learners' success in classroom settings. The literature has consistently demonstrated that motivation and personal interest are strong predictors of course performance [1, 2]. The expectancy‐value theory of motivation proposes that learner motivation is comprised of two components: The learner must find the learning important or valuable, and the learner must have a reasonable expectation of success [3]. For example, learners, who believe a particular learning activity has a high task value, tend to use deeper cognitive learning approaches, which result in longer term learning [4, 5, 6]. Similarly, there is a correlation between academic performance and learners' belief in their ability to succeed [7, 8, 9]. If one or both of these motivational components are absent, so too will be learners' motivation to complete a learning task [10]. As such, there has been an interest in developing teaching strategies to increase learner motivation by targeting these underlying intrinsic and extrinsic factors.

One strategy targeting learner motivation is the gamification of educational content [11, 12]. Gamification broadly speaking is the incorporation of game‐like elements into traditionally nongaming environments, in order to make an activity more fun or engaging [13, 14]. The use of game design elements such as points, leaderboards, performance graphs, and interactive components within educational spaces aims to increase learner engagement with an activity, resulting in increased perceived task value or task meaningfulness [15, 16, 17]. The clear goals and immediate feedback of gamified elements can act as scaffolds to improve learners' expectations of success [18]. Additionally, gamified elements can help learners build a more positive relationship with failure through the use of rapid feedback cycles, which explicitly make failure part of the learning process [19]. Gamified elements are often incorporated into preexisting teaching strategies, such as simulations and quizzes, to increase student engagement and enjoyment [16]. In the past 10 years, there has been increasing use of gamified interventions across postsecondary education, including humanities, health professions, engineering, physical science, and social science disciplines [20, 21, 22, 23, 24].

Gamification as an online teaching strategy dramatically increased during the COVID‐19 pandemic as a means to increase learner participation and motivation [25, 26]. A review of manuscripts evaluating gamification strategies employed during COVID‐19 found that learners perceived gamified activities as innovative, engaging, and helped them feel connection during pandemic isolation [27]. However, in some circumstances, learners felt overwhelmed by the pandemic, which led to reduced participation in gamified activities [28, 29].

Although the impact of gamified interventions in education is largely positive [30, 31], the delivery and administration of these interventions warrant further investigation. For example, learners report less motivation in gamified interventions where the educational content and game activities are disconnected, or game mechanics appear arbitrary when compared to conventional learning methods [32, 33]. As such, the technological delivery of gamified interventions can influence learners' outcomes and motivation. Similar research into delivery modality has found that motivation and learning outcomes increase as gamified interventions become more immersive [34]. Virtual reality (VR) is one modality of an immersive gamified intervention that has been shown to promote increased learning outcomes and motivation [35, 36, 37]. The use of virtual reality head‐mounted displays, also known as headsets, providing 360° immersion in simulations has been shown to be more effective than desktop virtual reality for most disciplines [35]. Virtual reality is frequently used in higher education to teach procedural or practical knowledge such as safety procedures, declarative knowledge such as anatomical names or key theoretical concepts, and analytical skills such as patient diagnosis or computer coding [38, 39]. Less frequent applications of VR in postsecondary education include team building, developing language skills, and developing behavioral learning [38]. Most literature on VR use in higher education focuses on health sciences, science, and engineering disciplines; however, there is a growing use of VR within arts and humanities contexts [39].

In this study, we examined how the use of VR headsets in a 3D VR laboratory simulation impact learner motivation and learning outcomes. We focused on virtual laboratory simulations available through Labster, a software company based in Denmark that develops interactive laboratory simulations that allow for open‐ended investigations [40]. The Labster platform consists of over 250 virtual laboratory simulations encompassing a wide range of STEM fields such as chemistry, biology, physics, anatomy and physiology, civil engineering and material science, earth and space science, biotechnology, ecology, and most recently nursing.

One of the main goals of Labster laboratory simulations is to engage students in specific areas of STEM by creating simulations that allow learners to conduct a virtual laboratory technique. In the era of hybrid learning, this type of online laboratory experience has been embraced by many universities, colleges, and high schools around the globe. The diversity of virtual laboratory simulations also allows educators to expose learners to equipment and techniques that would not be possible in a teaching laboratory setting. Such techniques are often cost‐prohibitive and inaccessible to learners in the physical environment of a laboratory.

The efficacy of desktop versions of Labster simulations has previously been examined, with learners demonstrating higher motivation, self‐efficacy, and learning outcomes compared with traditional lecture‐style instruction [41, 42]. Learners have also been shown to gain the same benefits from desktop Labster simulations whether they complete them at home or in a classroom environment [43]. As the use of virtual reality headsets has enhanced the positive effect of other gamified interventions, we asked how the immersive Labster simulations experienced with head‐mounted display technology would impact the learning and motivation of undergraduate students. Additionally, we asked learners about their ease of use with head‐mounted display technology. To our knowledge, this is one of the first assessments of using immersive virtual reality focusing on biomedical research techniques, such as polymerase chain reaction, next‐generation sequencing, transfection, and microscopy, within an undergraduate setting. Overall, our findings align with past research documenting gamified interventions increasing learner motivation and learning outcomes. This study also identifies best practices for the implementation of head‐mounted display technology in classrooms and laboratories.

## Methods

This study was evaluated by the Hamilton Integrated Research Ethics Board and determined to be exempted from ethics review, as its primary purpose was program evaluation. The main goals were to identify the specific effects of a given course intervention on students' interest and learning outcomes. Informed written consent was obtained from all participants during the program evaluation progress. Following program evaluation, secondary use of anonymized data was used for research purposes.

### Participant recruitment

**Fig. 1:**
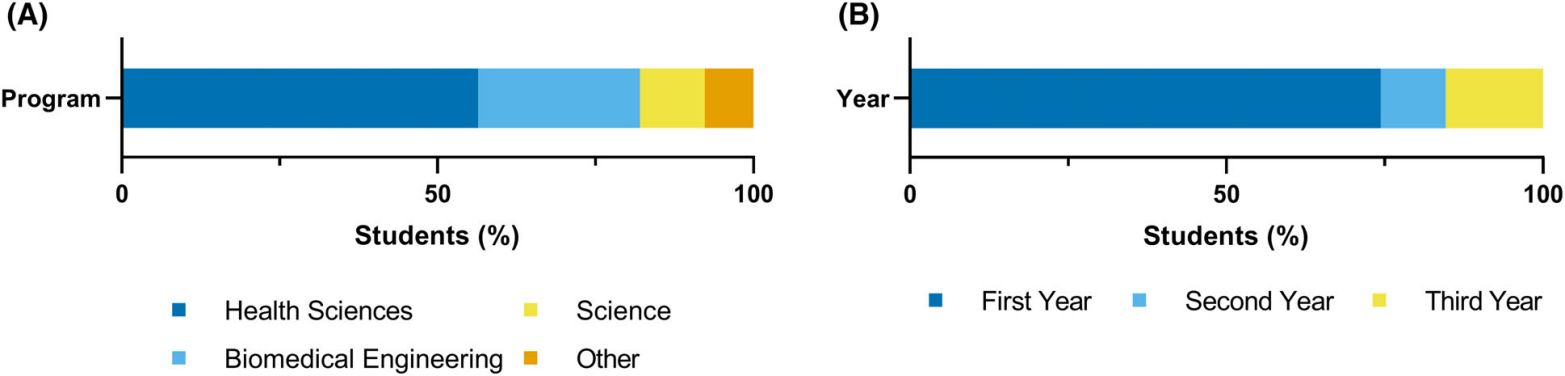
Participant demographics. (A) Participant program of study. Represented programs include health sciences, biomedical engineering, science (biochemistry, chemistry, general life sciences), and others (commerce, software engineering). (B) Participant program year of study.

The participant sample consisted of 39 undergraduate students from McMaster University in Hamilton, Ontario, Canada. Participants were recruited by online social media advertisements posted on student group pages. The majority of participants came from health sciences or biomedical engineering programs (82%) and were in their first year of study (74%) (Fig. 1). Participants were divided into two groups, completing either the Labster Gene Expression Unit simulation (*n* = 23) or the Viral Gene Therapy simulation (*n* = 16).

### Apparatus and simulation

**Fig. 2:**
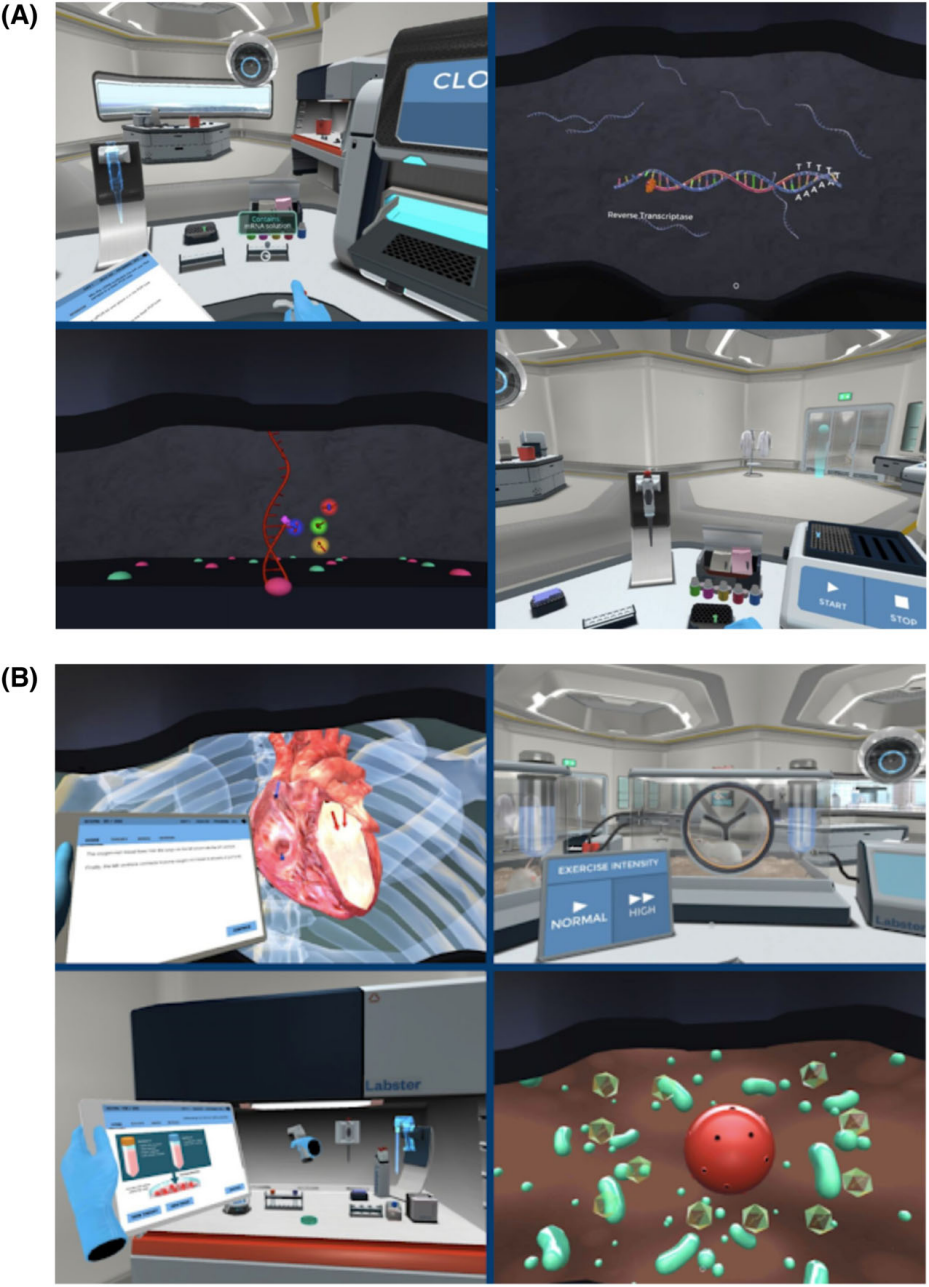
Labster virtual reality display. (A) Sample images from the Gene Expression Unit simulation. (B) Sample images from the Viral Gene Therapy simulation. ©Labster ApS 2022.

The Labster simulations were delivered using Google Daydream VR Headsets and controllers. Participants were able to adjust the headset straps and image clarity to their personal preference prior to beginning the simulation. The interface of the headset enclosed the eye area, allowing for a 360° immersive experience (Fig. 2). Participants were able to engage with content by turning their heads or body to examine the digital space, while using the controller to interact with objects.

The virtual laboratory simulations are constructed to incorporate a game‐like feel to a laboratory technique, which strategically amplifies the learner's experience. The learning outcomes of most Labster virtual laboratory simulations can be deconstructed to align with the 5E instructional model; which focuses on engagement, exploration, explanation, elaboration, and evaluation [44].

Most Labster virtual laboratory simulations present a laboratory technique in the context of a scenario. The scenario serves to not only capture the learner's interest but also place laboratory techniques in the context of a real‐life application. For example, in the polymerase chain reaction (PCR) laboratory simulation, the learner is immediately presented with a murder mystery. Upon collecting evidence, the learner enters a virtual laboratory space to conduct PCR, agarose gel electrophoresis, and DNA profiling. These data are then analyzed by the learner and the culprit is revealed.

In this study, participants completed either the Gene Expression Unit or the Viral Gene Therapy simulation. In the Gene Expression Unit simulation, learners are tasked with identifying a gene linked to obesity in pigs. Techniques depicted in this simulation include Next‐Generation Sequencing (NGS), generation of complementary DNA (cDNA), and quantitative PCR (qPCR). In the Viral Gene Therapy simulation, simulation learners are tasked with modifying a viral vector for use as a treatment for heart failure. Techniques depicted in this simulation include transfection of mammalian cells, production of viruses, and electron microscopy.

Each Labster simulation provides learners with an interactive LabPad when they enter the simulation. The interactive LabPad guides the learner through the simulation. The LabPad is the main console feature used to take the learner through the simulation. It provides instructions for the learner, and it allows the learner to explore their surroundings. The LabPad also contains a *Theory* tab that provides background information on specific concepts used in the simulation. The *Mission* tab often provides an overview of the completion tasks required in the simulation, and the *Media* tab can contain media used in the simulation itself. The learner can access the LabPad throughout the simulation.

The LabPad also has a *Score* and *Progress* bar. The *Score* bar pertains to multiple‐choice quizzes threaded throughout the simulation at specific points to solidify the main concepts presented in the laboratory simulation. The learner has multiple attempts for each quiz question, but a correct answer must be provided before the learner can advance in the simulation. Each incorrect quiz attempt decreases the maximum points earned for that question. Once the correct answer is selected, the Labster platform expands on the rationale for said answer. The *Progress* bar allows the learner to see what percentage of the simulation they completed. The simulations used in this study vary in duration from 37 min (viral gene therapy) to 74 min (gene expression), although these times are an approximation as student progress through each simulation is self‐paced.

### Pre‐ and postsimulation procedure

A pretest consisting of 10 multiple‐choice questions was administered to participants immediately prior to the VR simulation to assess participants' baseline content knowledge. After brief instructions on Labster technology use and Google Daydream VR Headsets adjustments, participants completed their assigned laboratory simulation guided by a virtual pedagogical assistant [45]. A posttest in a similar style and format as the pretest was administered to measure the gain in participant knowledge. The pre‐ and posttests assessed the same background concepts on gene expression and viral gene therapy and were of similar difficulty level.

The aim of the pre‐ and postsimulation tests was to gauge baseline content knowledge before the simulation (pretest) and content knowledge gained by completing the simulation (posttest). The rationale for the design of the pre‐ and posttests was inspired by the test‐enhanced learning theory published by Roediger and Karpickle [46]. We chose to use similar content on the pre‐ and posttests to aid in recognition and retrieval for each participant, which is a critical component of long‐term retention [47]. We also adapted content topics directly from the Labster simulations to enhance recognition and retrieval learning. In this way, the participant was initially assessed on their prior knowledge of the topic, they subsequently completed the simulation and were introduced to simulation‐specific content, and they were reassessed on content postsimulation.

For the gene expression simulation, the tests were aimed to assess general knowledge of techniques and terminology. The posttest was further refined to target the learner's understanding of specific techniques and data analysis. These questions are not simply recall‐based and require critical thinking skills. For the viral gene therapy simulation, the tests also aimed to assess general knowledge of the topics, with many of the posttest questions focused on viral gene therapy experimental design. In this way, the learner had to apply knowledge gained in the specific Labster case study to a more general understanding of these techniques.

### Measures

Following the testing process, participants provided feedback on their experience using Labster virtual reality with a Google Daydream VR Headset. This exit survey consisted of 18 questions which students completed in approximately 15 min. The survey had three sections: headset feedback, simulation feedback, and open‐ended feedback. Five‐point Likert scales were used to indicate headset device ease of use and incidence of adverse effects, such as eye strain, dizziness, motion sickness, and nausea. Using the previously published 4‐point Likert rating scale by Bonde et al., participants rated how VR technology and Labster simulations impacted their learning experience and motivation [41]. Finally, participants were given the opportunity to provide other feedback on the VR experience through an open‐ended question. Twenty‐seven participants (69%) chose to provide further feedback. These open‐ended responses were then coded and analyzed for themes via content analysis [48, 49]. We used a convergent mixed‐methods analysis approach, coalescing our quantitative and qualitative data to obtain a more holistic assessment of students' experiences [49].

### Statistical analysis

All statistical analyses were conducted in graphpad prism 8 (GraphPad Software, San Diego, CA, USA). We used the Shapiro–Wilk test to determined that both pretest and posttest dataset did not follow a normal distribution (Table 1). Prism robust regression and outlier removal (ROUT) method was used to identify one outlier in the posttest dataset. *P*‐values for paired datasets were calculated by the Wilcoxon matched‐pairs signed‐rank test. *P*‐values for unpaired datasets were calculated by the Mann–Whitney test.

**Table 1:** Tests of normality *P*‐values. All values were calculated using graphpad prism 8.

Shapiro–Wilk test	Pre‐test	Post‐test
*W*	0.9292	0.8325
*P*‐value	0.0169	< 0.0001
Passed normality test (alpha = 0.05)?	No	No
*P*‐value summary	*	****

## Results

**Fig. 3:**
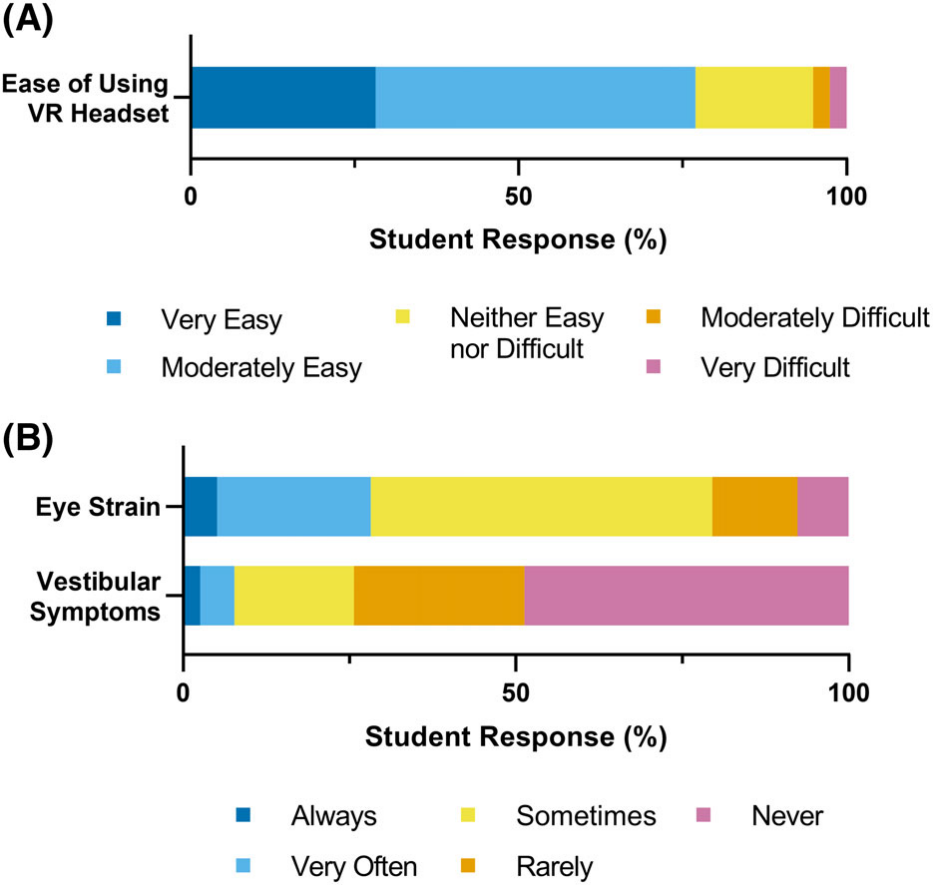
Feedback on virtual reality headset system. (A) Learner rating of system ease of use (*n* = 39). (B) Frequency of adverse effects while using the headset, including eye strain and vestibular symptoms (dizziness, motion sickness, or nausea) (*n* = 39).

First, we assessed the participants' experience using the VR headset equipment. The majority of participants (77%) rated the headset “very easy” or “moderately easy” to use (Fig. 3A). The most commonly reported adverse effect while using the system was eye strain (Fig. 3B). Half of all participants reported experiencing eye strain “sometimes,” (51%) and 28% reported symptoms “very often” or “always” (Fig. 3B). More serious vestibular symptoms, such as dizziness, motion sickness, or nausea, happened less frequently with 74% of participants “rarely” or “never” having them occur (Fig. 3B). Analysis of qualitative feedback also uncovered that approximately 30% of respondents reported that the heaviness of the VR headset contributed to neck strain, headaches, and general discomfort. The incidence of these symptoms was tied to prolonged use of the device:

Only issue was the physical weight of the headset, which might be burdensome in a longer lab simulation.

The only big concern was using the headset with glasses, since the headset was relatively heavy, which put pressure on my glasses and face, making it a bit uncomfortable towards the end.

These findings suggest that in future VR laboratory experiences, it could be beneficial to have scheduled breaks with the experience to reduce the incidence of such symptoms. Alternatively, VR headset equipment that weighs less could be implemented.

**Fig. 4:**
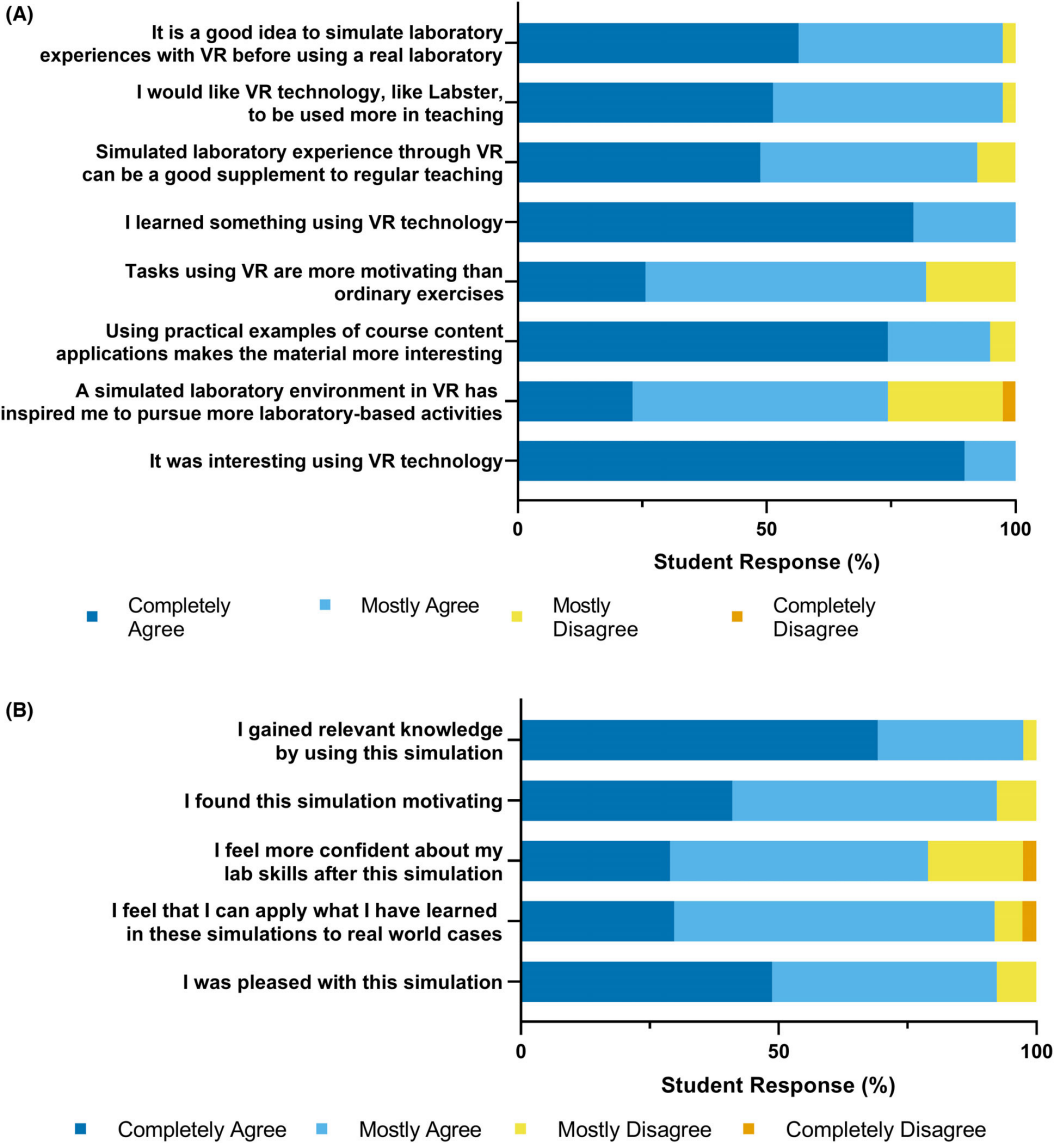
Learner rating of virtual reality experience postsimulation. (A) Learner rating of virtual reality on their learning experience and motivation (*n* = 39). (B) Learner rating of Labster simulations on their learning experience and motivation (*n* = 37–39).

Next, we investigated whether the use of virtual laboratory simulations increased participant motivation to learn the material and improved their learning experience. Participant views were highly favorable of VR, with 82% indicating VR exercises were more motivating than traditional learning exercises and 97% wishing VR technology was implemented more often in classroom teaching (Fig. 4A). Additionally, 91% of participants thought VR laboratory simulation would be a good supplement to regular teaching (Fig. 4A). There were sentiments that VR simulations “would not be able to replicate in person labs” and that simulations could be “too long and detailed for people without background knowledge to follow.” These statements further reinforce the careful and intentional incorporation of VR simulation technology into the curriculum. The learners' feedback suggests that VR simulation technology could serve to complement traditional laboratory teaching, rather than replace it entirely.

We also asked participants to rate their impressions of the Labster simulations they experienced. Similar to their rating of VR, the simulations themselves were viewed extremely positively, with 97% of participants indicating that they gained relevant knowledge from the simulation (Fig. 4B). Furthermore, 92% of participants reported that they found the simulation motivating (Fig. 4B). Written feedback also suggested that this motivation could stem from the “fast‐paced” nature of the simulations and the opportunity to conduct unique experiments:

I found the experiment very useful and am intrigued by the opportunities this method offers, such as shortening long experiments or the ability to do expensive labs that wouldn't have been possible in the real world.

Minor technical issues were reported with the simulations, including text being too small to read and issues selecting items. Overall though, 91% of participants were satisfied with the Labster simulations (Fig. 4B). A few participants commented that the simulations were too restrictive, with people unable to make mistakes or complete incorrect actions:

At some points, I tried to do things outside of what the experiment told me to see if I could do the lab incorrectly. Unfortunately, the simulation didn't let me make mistakes that affect the result of the experiment.

This suggests these participants were looking for an experience where they could learn through trial and error, as opposed to the structured format currently employed by Labster. In the current format, students can only proceed by completing steps correctly. This warrants further consideration when designing future simulation experiences that might better recapitulate in‐person laboratory experimentation. Although Labster simulations do not perfectly recapitulate a wet laboratory setting, there are still benefits to be gleaned from completing simulations. Completing simulations, particularly as a prelaboratory exercise, can help students gain familiarity with steps, procedures, and equipment which can improve confidence in executing laboratory techniques [50, 51].

**Fig. 5:**
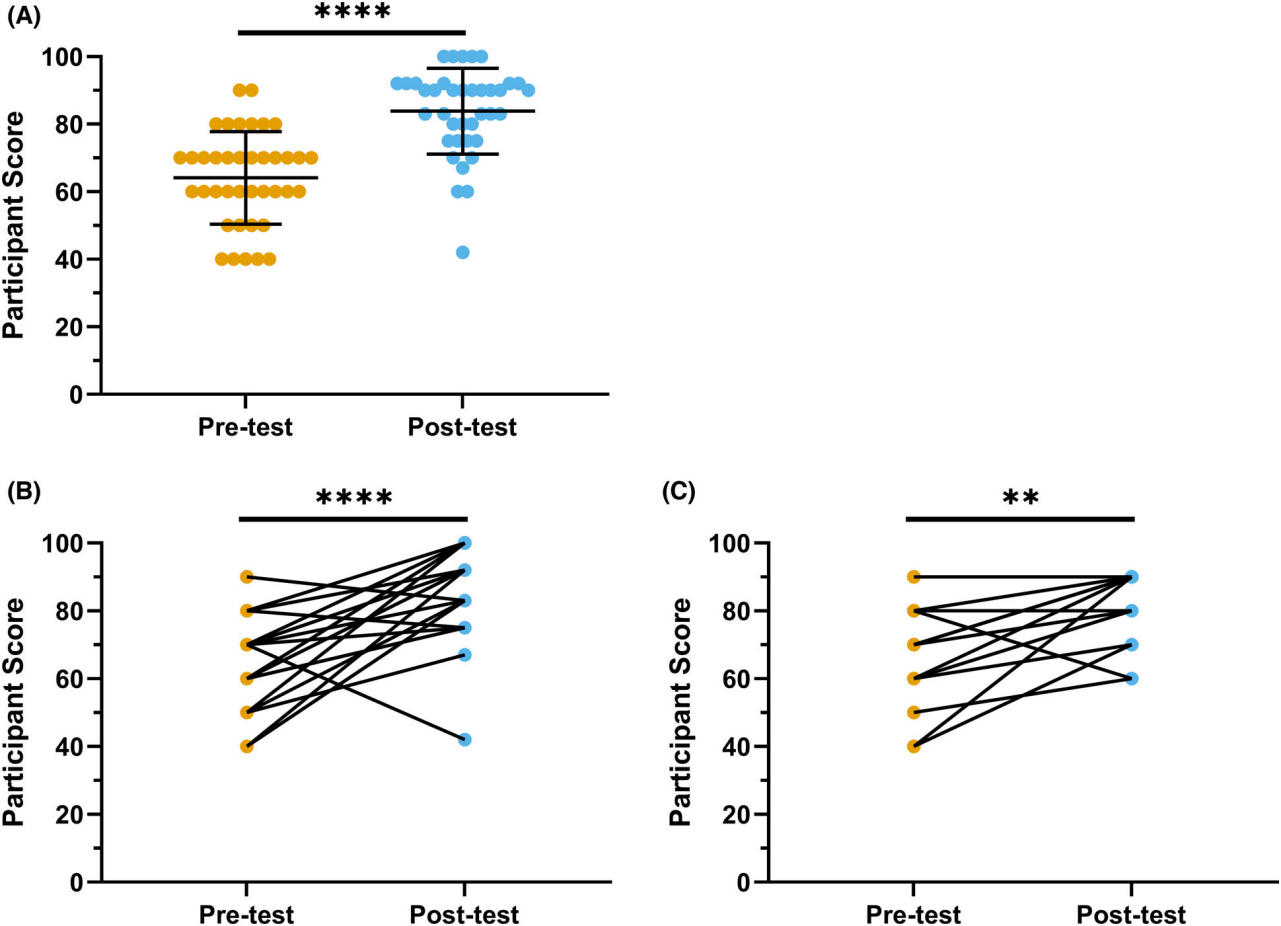
Measurement of learning outcomes pre‐ and postsimulation. Significance values calculated by Wilcoxon matched‐pairs signed‐rank test. **P ≤ 0.0027, ****P ≤ 0.0001. (A) Test outcomes of total learner population (*n* = 38). Outliers were identified by GraphPad Prism ROUT test. (B) Test outcomes of learners in gene expression unit simulation (*n* = 22). (C) Test outcomes of learners in Viral gene therapy simulation (*n* = 16).

**Fig. 6:**
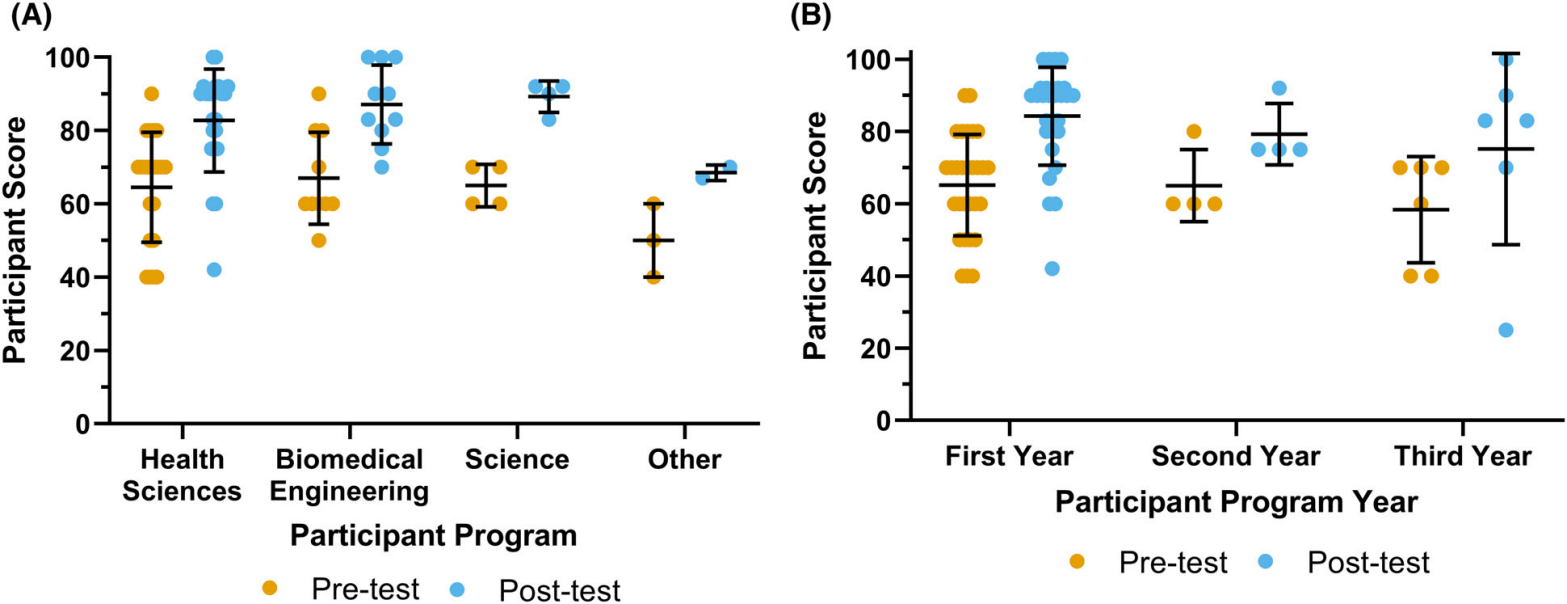
Breakdown of participant score based on demographics. No statistically significant patterns were identified based on the participant program or year of study. Error bars represent standard deviation and mean. Significance values calculated by Wilcoxon matched‐pairs signed‐rank test. (A) Participant score divided by program of study. (B) Participant score divided by year of study.

Lastly, we examined how the use of virtual simulations impacted participant learning outcomes. Prior to completing the simulations, the average test score of participants was 64.1% (Fig. 5A). There were no significant differences in test scores between the Viral Gene Therapy (66.3%) and the Gene Expression Unit simulation (62.7%) learner populations (Fig. 5B,C). Following the use of VR, the average test score significantly increased to 82.3% (Fig. 5A). Test scores for the Viral Gene Therapy and Gene Expression Unit simulation raised to 81.9% and 82.2%, respectively (Fig. 5B,C). There was no significant impact of the learner's program or year of completion on test scores before or after the simulation (Fig. 6). These results are consistent with previously reported finding that learning outcomes improve following the use of VR simulations [41]. Future studies will be needed to investigate whether how immersive 3D simulations impact learning outcomes as compared to other interventions; such as a lecture, assigned readings, or 2D laboratory simulation.

## Discussion

In this study, we assessed the impact of head‐mounted display‐mediated gamified laboratory simulations on learners' motivation and learning outcomes. We used a mixed‐methods analysis approach with pre‐ and postsimulation knowledge testing data, along with postsimulation experience surveys of 39 undergraduate learners. The majority of participants reported the virtual reality headsets were easy to use. While eye strain was a common occurrence, more serious vestibular side effects that might preclude the use of headsets or students' ability to complete the simulations use were uncommon. Consistent with previous literature, our participants had significantly improved test scores following the gamified intervention [41, 42, 52, 53]. Postsimulation survey responses indicate high levels of motivation and engagement with the gamified experience, which aligns with past data on Labster and other gamified laboratory simulations [41, 42, 43]. Our findings support that immersive VR laboratory simulations with head‐mounted display technology have positive outcomes similar to desktop simulations. To the best of our knowledge, this is the first study to analyze 3D VR simulations of biomedical research techniques. While there are ample data supporting the use of 2D simulations, there is a paucity of data on immersive simulations such as the ones investigated in this study.

We found no significant differences in the increase of scores between the two laboratory simulations examined. This suggests that the modality of the intervention, not the content itself, impacted the motivation and engagement of participants. Furthermore, we found no differences in learning outcomes or motivation between participants from different program levels or fields of study. This is consistent with previous findings which suggest that this style of immersive simulations has the potential to enhance learning regardless of previous experience or disciplinary background knowledge [38, 39]. Participants reported that they felt they gained relevant knowledge following the use of the simulation. This includes learners who indicated that without these simulated experiences, they would not have the opportunity to have similar in‐person laboratory experiences.

Another strength of our study was the identification of best practices for the implementation of 3D VR simulations. Similar to previous examinations of head‐mounted displays, our participants reported that the headsets were easy to use, with minimal adverse effects [53, 54]. Eye strain was the most commonly observed adverse effect, which can be mitigated through scheduled breaks [53, 55]. Some participants indicated the weight of the Google Daydream VR headset was an issue following prolonged use. Symptoms associated with weight, such as neck strain and headaches, could be lessened through scheduled breaks, or the use of a lighter head‐mounted device. There were also minor technical reports about the Labster interface, including text that was too small and items that were difficult to select. Almost all participants were satisfied with the content of the simulation overall. This indicates that these technical difficulties were not a major difficulty for most participants. Nevertheless, over three‐quarters of participants reporting eye strain and a quarter reporting vestibular side effect pose a serious issue for future implementation of VR technology. Other groups developing VR laboratory simulation have reported similar side effects in participants following prolong VR headset use [56, 57]. This points to the need for future inquiry into strategies for reducing eye strain and vestibular symptoms, such as intermittent breaks or reducing motion of visual stimuli [58], prior to widespread adoption.

One limitation of our analysis is we did not collect information on participants' gender, which has been linked with the incidence of vestibular symptoms [59, 60]. As women have been reported to experience motion sickness more frequently while using head‐mounted displays, this information could have been factored into our analysis [59, 60]. Another limitation is that we did not reassess participants following the study to measure the retention of knowledge over time. Work by other groups indicates that VR simulations may improve long‐term knowledge retention [61, 62]. Lastly, this work relied on self‐reported measures, which can be impacted by social desirability bias [63]. We used an anonymous survey to minimize the impact of social desirability bias, along with the option to not provide potentially identifying qualitative responses.

While our study and others highlight the benefits of immersive simulations such as higher engagement and motivation, there are additional considerations in the implementation of these in a laboratory or classroom setting. Each Google Daydream Headset costs ~ $400 USD, which presents challenges in scaling immersive headsets for large courses. This could be overcome through rotating groups through the simulations or implementing a mix of 2D and immersive simulations. From an instructor perspective, an additional benefit of students completing 2D simulations is the ability to provide feedback to students in real‐time. This is a trade‐off of the self‐contained immersive headsets, where the instructor is unable follow along the simulation with the student and reinforces thoughtful incorporation of curricular elements.

## Conclusion

Overall, our results support further exploration of combining gamified VR laboratory simulations with conventional laboratory teaching. Participants expressed that although simulated experiences were beneficial, they could not replicate the variability and overall experience of in‐person laboratory training. As suggested by other groups [64], the combination of in‐person laboratory teaching supplemented with VR simulations may be the optimal strategy for using these gamified interventions. Additionally, VR simulations with head‐mounted devices could make laboratory learning more accessible to learners who are unable to physically attend in person [65].

Further research is needed to assess the most favorable way to implement laboratory simulation alongside in‐person training. More specifically, future lines of inquiry could include timing (pre or post in‐person laboratory) of virtual interventions, accessibility considerations, cost of implementation, and comparing the benefits of 2D versus immersive simulations. Overall, our results add to a growing body of evidence that gamified laboratory simulations can enhance learning outcomes and increase motivation. Virtual reality simulations such as these have the potential to become an advantageous learning tool in the classroom and laboratory.

## Conflict of interest

The authors declare no conflict of interest.

## Author contributions

FV and CM conceived of the study. FV, CM, DT, and CS participated in the design. DT collected the data as described in the methods. CS analyzed and interpreted the data. CS and RA wrote the first draft, with FV and CM providing revisions.

### Peer review

The peer review history for this article is available at https://publons.com/publon/10.1002/2211‐5463.13567.

## Data Availability

As this study reflects the secondary use of program evaluation data, the data are available only by request to the corresponding author.
